# Improved control of non-communicable diseases (NCDs) requires an additional advanced concept for public health – a perspective from a middle-income country

**DOI:** 10.12688/f1000research.18423.1

**Published:** 2019-03-14

**Authors:** Benja Muktabhant, Frank Peter Schelp, Ratthaphol Kraiklang, Pornpimon Chupanit, Pattara Sanchaisuriya

**Affiliations:** 1 Faculty of Public Health, Khon Kaen University, Khon Kaen, Thailand

**Keywords:** public health, non-communicable diseases, sustainable development goals, primary health care, human metabolism, molecular epidemiology, epigenetic, geroscience, healthy aging

## Abstract

A major consequence of all elements of the ‘epidemiological transition’ is the rapid emergence of non-communicable diseases (NCDs) in low- and middle-income countries. In contrast to the outcomes of the ‘Alma Ata Conference for Primary Health Care’, it has not yet been possible to introduce an equally powerful health policy for the prevention and control of NCDs. Major strategies so far are to advise individuals not to smoke and drink alcohol in excess. Additionally, ‘healthy’ nutrition and increased physical activity are also advocated. Policy for preventing and working against NCDs is now part of the Sustainable Development Goals, specifically target 3.4. So far, attempts to soften the influence of NCDs on the health of the people in low- and middle-income countries have been unsuccessful. It is argued here that additional concepts on how public health could operate against NCDs are needed.  Major risk factors for NCDs interfere with and alter complex steps within the human metabolism.  This paper explores how human metabolism works by assessing advances in molecular biology and research in genetics, epigenetics and gerontology. Recent developments in these scientific disciplines shed light on the complexity of how human health is maintained and diseases are invoked. Public health bodies should be aware, interested and possibly contribute to the aforementioned areas of interest, as far as NCDs are concerned, and translate major developments in a way, that could be useful in improving population health.

## Background

In recent decades, mortality due to non-communicable diseases (NCDs) have become priority health problems in countries in South and South-East Asia, as well as Latin America and Africa ^1^. From the perspective of high-income countries ^2^, this development did not fit well with infectious diseases, previously thought to be one of the issues for those countries ^3^, which are predominantly located in the tropics and sub tropics. Many of these formerly so-called ‘developing countries’ still have low technical and financial resources. Their geographical location implies an environment favorable for infectious diseases. Although a number of infectious diseases are linked to NCDs ^4^ at least to develop strategies to control the spread of infective agents, through various means such as hygienic measures, impregnated bed nets against malaria, antibiotics etc., is relatively easy in comparison to changing the ‘unhealthy behavior’ of individuals ^5^. It is well accepted that ‘unhealthy behavior’ is the main underlying cause for the occurrence of NCDs.

NCDs have been growing in high-income countries for much longer than in low and middle-income countries. In most high-income countries, the majority of the population lives an urban lifestyle, even if residing in rural areas. Under these circumstances’ public health bodies concentrate on how to finance health care and use mass media for health messages. Low- and middle-income countries base their health delivery systems on public health strategies focused on rural areas ^6^, in which the majority of the population lives, while urban area lifestyles differ significantly from rural ones ^7^. In rural areas it was and still is relatively easy for public health personal to approach the population directly.

The living conditions and the economic situation in many low- and middle-income countries have improved considerably in recent decades. However, public health initiatives of low- and middle-income countries cannot simply adopt the ways and means of high-income countries to cope with NCDs, but have to adjust public health policies to their own biological, socio-economic and cultural environments. It is argued in this communication that present strategies of public health, from low-, but in particular middle-income countries, should be reshaped by integrating developments and results in molecular biology, genetics and epigenetics as well as attempts to enable ‘healthy aging’ to facilitate the long-term control and prevention of NCDs.

## Primary health care

Low- and middle-income countries orientate the formulation of their health policy on recommendations from institutions of the United Nations. The World Health Organization (WHO) plays a crucial role in providing expertise, and assisting in setting targets scenarios for health policies of global importance. In 1978, WHO and United Nations International Children's Emergency Fund (UNICEF) were the leading UN agencies at the International Conference on Primary Health Care held in Alma-Ata, at that time the capital of Kazakhstan, then part of the Soviet Union. The major outcome of the conference was the Alma-Ata Declaration, which shaped health policies for decades worldwide. Key issues of primary health care (PHC) was the quest for a ‘comprehensive, universal, equitable and affordable health care service’ with the involvement of the community ^8^. The original concept of ‘health for all by the year 2000’ was too ambitious ^9^ and PHC in most low- and middle income countries tried to concentrate further on mother and child health care (MCH), undernutrition, hygiene, and prevention of infectious diseases ^10^. Thirty years after the Alma Ata Declaration the usefulness of PHC was shown in the reduction of child mortality, increase in immunization and family planning for a number of countries. Especially Thailand was mentioned in achieving the highest average yearly reduction in mortality among the ‘under-fives’ ^11^.

Unfortunately, the success of PHC was obscured by the emergence of AIDS and NCDs. Public health authorities started to face a ‘double burden’, providing care for infectious diseases but also facing the rapid increasing incidence and prevalence of NCDs ^1^. The emergence of NCDs is often referred to as ‘the epidemiological transition’ including the ‘demographic-‘ and ‘nutritional transition’ ^12^. These developments were due to improved food security, prevention and advanced treatment of infectious diseases, as well as improvements in medical care as a whole and reduced fertility. Yet financial donors, who in many countries support the health system, were slow to acknowledge that NCDs were increasingly becoming a problem for poorer countries. This might be accounted for by the fact that controlling and treating infectious diseases, at least conceptually, is easier than preventing and caring for patients with NCDs. Communicable diseases are usually caused by one particular agent causing a disease with limited duration. Once the mode of infection and the life cycle of the infective agent are known, preventive and control measures can often be developed much more easily in comparison with NCDs. In contrast, NCDs have started to occur in low- and middle-income countries ‘ with lethal consequences’, particularly in poorer countries where the health delivery system continued to focus heavily on infectious diseases, with little or no priority given to NCDs.

## Sustainable Development Goals (SDG)

The pressure to pay adequate attention to the obvious problem which NCDs pose to low- and middle-income countries has finally become a political issue of global importance. The countries exposed to the phenomenon of the ‘epidemiological transition’ were well aware of the problem ^12^. For instance at the start of the millennium NCDs were of major concern for the Thai Ministry of Public Health ^13^. Initiated by Bangladesh and Caribbean Communities and supported by the International Diabetes Federation (IDF) ^14^, it was finally decided that a ‘High Level Meeting’ on NCDs should be held September 19 to 20, 2011 in New York. Major additional driving forces for the meeting was the Global NCD Alliance and the ‘Lancet NCD Action’ ^15^. As far as prevention was concerned, the focus was laid on the so-called ‘ best buys’: Improve tobacco control, eliminate tobacco use; reduce salt intake; promote diets low in fats and sugar; reduce alcohol consumption; increase physical activity; and treatment with generic drugs ^16^.

The 2011 meeting was followed by a meeting in 2014 which established the most recent basis for a global policy known as the SDGs. Under target 3.4, the SDGs focus on cardiovascular diseases, diabetes, chronic obstructive pulmonary diseases and cancer ^17^. The aim is to decrease premature death through NCDs by one-third up to 2030. The four main risk factors mentioned are smoking, ‘unhealthy’ diets, physical inactivity and ‘harmful’ use of alcohol. Governments and public health authorities are encouraged to enforce so called 16 ‘best buy’ strategies to reach the target.

## Poor prospects to reach SDG’s targets for controlling NCDs

In preparation of another UN High-Level-Meeting in 2018, it was realized that no progress in adequately dealing with NCDs was achieved and also could not be expected up to 2030, unless drastic action from governments was forthcoming. In preparation for the 2018 meeting the ‘Lancet Taskforce for NCDs and economics’ suggested to ‘use fiscal policies to regulate the prices and consumption of potential unhealthy products. The main targets are tobacco, alcohol, soft drinks and snacks ^18^. However, the intention to stimulate governments to increase taxes in particularly on sugar and soft drinks failed and it was concluded that the ‘ high-profile, somewhat risky and ultimately sobering test of the proposition that non-communicable disorders (NCDs) could become a new global health priority’. Feasible suggestions to governments were to increase taxes on tobacco, eliminate ‘trans fats’, work against consumption of salt, sugar and saturated fats and increase physical activity. Screening, counseling and therapy for heart disease and cervical cancer was outlined as well as immunization against hepatitis B.

## Present strategies and problems of public health to work against NCDs

The disappointing overall results of the meeting, despite the forceful attempts of influential bodies such as the NCD Alliance and the Lancet Taskforce on NCDs ^18^, should not have been unexpected. To increase taxes on very popular consumer products prompts counteractions of the commercial sector ^19^. A telling example is how the US beverage manufacturers campaigned against cities willing to increase local taxes on sugar and soft drinks, by lobbying against statewide legislation via campaigns against ‘tax happy politicians’. Although tax increases were not planned for any grocery articles, except sugary beverages, the initiative to increase taxes on these items was finally abandoned ^20^.

Despite obvious constrains, the need and usefulness to work on health policies and agree on overall targets to improve world-wide developments cannot be questioned. It is the duty of governments and respective ministries to push forward towards the wellbeing of the populations under their responsibility. To work for prevention of NCDs remains a difficult task for governmental agencies, which already face difficulties providing an affordable health delivery system for those already suffering from NCDs. The main risk factors for a number of important NCDs are related to what is inhaled, as far as smoking is concerned, and ingested by mouth, in terms of alcohol and nutrients. People are generally more afraid of the more immediate and obvious effects of infectious diseases and hardly think about NCDs, which might or might not, affect them in the distant future ^21^. To regulate behavior in authoritative ways creates resistance. The social sciences try their best to influence behavior based on well thought out but rather complex theories, such as the ‘Health Belief Model’, ‘Trans Theoretical Model’, and the ‘Theory of Planned Behavior’ and more recently by ‘Health Literacy’ ^22– 24^.

## Contemplating the complexity of human metabolism

It is possible that ‘health education’ could be more sustainable and more convincing, especially for educated, highly motivated, health conscious individuals, if the complexity of the human metabolism is given more attention by public health authorities with regards to health-related suggestions. This should also include the influence of genetic factors, the interaction of the environment on inherited factors, and the developments in the search for indicators measuring biological age, which might, in a positive way, trail chronological age. All this should be communicated in a way that is easily comprehensible by the general public. Governmental health authorities should consider, plan and monitor developments in health and diseases more efficiently, if the aforementioned issues are to be better known and understood. Those responsible for the improvement of the health of communities, on the professional and academic level, should be the link between medicine as well as molecular biology to public health, to turn recent advantages in epigenetic, and ‘gerosciences’ to the benefit of overall populations. This could be done without giving up the achievements of public health in following the strategies of PHC in the past, but could be even more constructive by integrating additional new concepts as it is mentioned here.

## Population based research in molecular epidemiology and other biomedical fields

To a certain extent, population-based research applying the tools of molecular epidemiology ^25^, is already done of public health institutions and those representing other areas, such as medicine, nursing, social sciences and tropical medicine. In a subsequent communication the subject of molecular epidemiology and the use of biomarkers will be discussed more specifically. Additionally, we argue, increased attention should be given to the interaction of diseases such as diabetes and cancer ^26– 28^, genetic sciences, epigenetic features and geroscience.

Carriers of certain genes are at risk of obesity ^29, 30^, and others develop diabetes mellitus and cancer ^31, 32^. Studies so far are more or less restricted to populations of high-income countries ^33^. The importance of genetic factors for the manifestation of diabetes and cancer are not yet well known but should also be considered as potential important risk factors as well in addition to environmental risks especially for low- and middle-income countries.

Epigenetic mechanisms are even more of importance. Nessa Carey, in her book ‘the Epigenetics Revolution’ (2012) ^34^ mentioned that in the nineteenth century, Darwin and Mendel defined the area of evolution and genetics, Watson and Crick in the twentieth century the area of DNA, and in the twenty-first century ‘ *the new scientific discipline of epigenetics’* …[deconstructed]… *’so much of what we took as dogma and...*[rebuild a]… *’more complex, and…more beautiful fashion*.’

Epigenetics was originally defined as the interaction between genetics and environment. A more accurate definition refers to gene functionality which is not encoded into the DNA sequence, but can be hereditary. That is, the code of the DNA is not changed but the transcription from the code. One of the major control mechanisms related to epigenetics is linked to DNA methylation, for example the addition of a methyl group at the 5th carbon of the cytosine base at a CpG dinucleotide pair. Other mechanisms of epigenetic modification are chromatin remodeling and micro-RNAs modifying gene expression ^35^. The important concept here is that the function of the gene is modified by environmental influences, especially in the field of nutrition ^36^. Of particular interest for public health should be epigenetic features in embryogenesis and postnatal developments related to gestational diabetes, and efforts to prevent non-communicable diseases in later life or even in future generations. These aspects are of critical potential importance for mother and child health services ^37, 38^.

## Geroscience and healthy aging

Epigenetic functions also play a major role in aging. The so called ‘epigenetic clock’ ^39^ received much attention in the attempt to differentiate the ‘biological age’ from the ‘chronological age’, an active research field in ‘geroscience’, formerly called ‘biogerontology’ ^40^. Research on biological aging tries to answer the question of why some people are already rapidly aging in their fifties, while others appear very young in mind and active far above their 80th birthday ^41, 42^. ‘Healthy aging’ is of eminent importance, since in the context of the demographic transition the elderly population is already increasing in low- and middle-income countries. The reasons for this include improved medical care, decrease in infectious diseases, better mother and child health care, better nutrition, hygiene, housing and education. All these combine to produce higher life expectancy ^43– 45^. While research in ‘geroscience’ manipulates the genetic settings of laboratory animals and the results are not yet of relevance for public health; preventive medicine, however, should work for ‘healthy aging’, and in this respect geroscience is of particular interest for public health. Increasing age, as such, is a risk factor for NCDs ^46^. In future the topic ‘healthy aging’ will not only be of high relevance for high income countries but a global aspiration.

## Summary and conclusion

Prevention and control of NCDs requires establishing among the population generally ‘healthy’ behaviors. Conservative public health strategies should be supplemented to promote longer lasting ‘intrinsic’ motivation for change into healthier life styles. This requires the complexity of human metabolism to be well communicated by health authorities and well understood by the recipients of the message. A precondition for this is that health authorities are more aware of developments in molecular biology, genetics and epigenetic features. In the near future it will be indispensable to work for ‘healthy aging’ by means of public health initiatives. It will be of mutual benefit for players in molecular biology, medicine, public health and governmental health authorities and other related fields to cooperate more closely. Cooperation doesn’t mean solely to support or join together in research projects and make use of the results for improving the health of the population, it also means a willingness for all involved to look beyond the narrow boundaries of their fields of interest.

## Data availability

No data are associated with this article.
