# Supplementary material for: Improved control of non-communicable diseases (NCDs) requires an additional advanced concept for public health – a perspective from a middle-income country
Source: F1000Res. 2019 Mar 14;8:286. [Version 1] doi: 10.12688/f1000research.18423.1 (PMC6685454; doi:10.12688/f1000research.18423.1)
Supplement: Peer review history

# Peer review: Reviewer response for version 1

**DOI:** 10.5256/f1000research.20157.r48826

**Published:** 2019-08-06

**Authors:** Tanida Phatisena

**Affiliations:** 1 Faculty of Public Health, Nakhon Ratchasima Rajabhat University, Nakhon Ratchasima, Thailand

This article shows the opinions and the problems of NCD which is a rising trend. The authors analyse about the determinant of chronic disease thoroughly. This article is interesting because the authors show the main point of the factor of genetics which causes NCD. Therefore, health authorities should aware of developments in molecular biology and epigenetics for healthy aging.

I confirm that I have read this submission and believe that I have an appropriate level of expertise to confirm that it is of an acceptable scientific standard.

# Peer review: Reviewer response for version 1

**DOI:** 10.5256/f1000research.20157.r45728

**Published:** 2019-04-17

**Authors:** Frank P. Mockenhaupt

**Affiliations:** 1 Institute of Tropical Medicine and International Health, Charité - University Medicine Berlin, Berlin, Germany

This is a thoughtful piece of opinion highlighting the need for additional efforts of Public Health systems in LMICs to achieve improved prevention and management of NCDs. The authors call for a better integration of various disciplines and consideration of epigentics and "healthy ageing".

 Beyond some editing, this paper can be indexed as it stands.

I confirm that I have read this submission and believe that I have an appropriate level of expertise to confirm that it is of an acceptable scientific standard.
